# Prediction of early improvement of major depressive disorder to antidepressant medication in adolescents with radiomics analysis after ComBat harmonization based on multiscale structural MRI

**DOI:** 10.1186/s12888-023-04966-8

**Published:** 2023-06-26

**Authors:** Huan Ma, Dafu Zhang, Yao Wang, Yingying Ding, Jianzhong Yang, Kun Li

**Affiliations:** 1 Department of Radiology, The Third Affiliated Hospital of Kunming Medical University, Kunming, 650018 China; 2 Department of Psychiatry, The Second Affiliated Hospital of Kunming Medical University, Kunming, 650101 China

**Keywords:** Major depressive disorder, Antidepressant medication, Magnetic resonance imaging, Radiomics, Machine learning

## Abstract

**Background:**

Due to individual differences and lack of objective biomarkers, only 30-40% patients with major depressive disorder (MDD) achieve remission after initial antidepressant medication (ADM). We aimed to employ radiomics analysis after ComBat harmonization to predict early improvement to ADM in adolescents with MDD by using brain multiscale structural MRI (sMRI) and identify the radiomics features with high prediction power for selection of selective serotonin reuptake inhibitors (SSRIs) and serotonin norepinephrine reuptake inhibitors (SNRIs).

**Methods:**

121 MDD patients were recruited for brain sMRI, including three-dimensional T1 weighted imaging (3D-T_1_WI)and diffusion tensor imaging (DTI). After receiving SSRIs or SNRIs for 2 weeks, the subjects were divided into ADM improvers (SSRIs improvers and SNRIs improvers) and non-improvers according to reduction rate of the Hamilton Depression Rating Scale, 17 item (HAM-D_17_) score. Then, sMRI data were preprocessed, and conventional imaging indicators and radiomics features of gray matter (GM) based on surface-based morphology (SBM) and voxel-based morphology (VBM) and diffusion properties of white matter (WM) were extracted and harmonized with ComBat harmonization. Two-level reduction strategy with analysis of variance (ANOVA) and recursive feature elimination (RFE) was utilized sequentially to decrease high-dimensional features. Support vector machine with radial basis function kernel (RBF-SVM) was used to integrate multiscale sMRI features to construct models for early improvement prediction. Area under the curve (AUC), accuracy, sensitivity, and specificity based on the leave-one-out cross-validation (LOO-CV) and receiver operating characteristic (ROC) curve analysis were calculated to evaluate the model performance. Permutation tests were used for assessing the generalization rate.

**Results:**

After 2-week ADM, 121 patients were divided into 67 ADM improvers (31 SSRIs improvers and 36 SNRIs improvers) and 54 ADM non-improvers. After two-level dimensionality reduction, 8 conventional indicators (2 VBM-based features and 6 diffusion features) and 49 radiomics features (16 VBM-based features and 33 diffusion features) were selected. The overall accuracy of RBF-SVM models based on conventional indicators and radiomics features was 74.80% and 88.19%. The radiomics model achieved the AUC, sensitivity, specificity, and accuracy of 0.889, 91.2%, 80.1% and 85.1%, 0.954, 89.2%, 87.4% and 88.5%, 0.942, 91.9%, 82.5% and 86.8% for predicting ADM improvers, SSRIs improvers and SNRIs improvers, respectively. *P* value of permutation tests were less than 0.001. The radiomics features predicting ADM improver were mainly located in the hippocampus, medial orbitofrontal gyrus, anterior cingulate gyrus, cerebellum (lobule vii-b), body of corpus callosum, etc. The radiomics features predicting SSRIs improver were primarily distributed in hippocampus, amygdala, inferior temporal gyrus, thalamus, cerebellum (lobule vi), fornix, cerebellar peduncle, etc. The radiomics features predicting SNRIs improver were primarily located in the medial orbitofrontal cortex, anterior cingulate gyrus, ventral striatum, corpus callosum, etc.

**Conclusions:**

These findings suggest the radiomics analysis based on brain multiscale sMRI after ComBat harmonization could effectively predict the early improvement of ADM in adolescent MDD patients with a high accuracy, which was superior to the model based on the conventional indicators. The radiomics features with high prediction power may help for the individual selection of SSRIs and SNRIs.

**Supplementary Information:**

The online version contains supplementary material available at 10.1186/s12888-023-04966-8.

## Background

Major depressive disorder (MDD) is the leading cause of disability in adolescents and the incidence increases dramatically during adolescence [1]. Approximately 70% of adolescents with MDD will relapse within five years, and early-onset depression is also associated with high recurrence rates, poor functional outcomes, and refractory depression in later years [2, 3]. Therefore, there is no doubt about the importance of early and proactive treatment for adolescent MDD patients. Antidepressant medication (ADM) remains the preferred treatment strategy for MDD, and clinical guidelines recommend the selective serotonin reuptake inhibitors (SSRIs) or 5-hydroxytryptamine and serotonin norepinephrine reuptake inhibitors (SNRIs) as the first-line antidepressant agents [4, 5]. However, due to individual differences in patients with MDD, less than 50% of patients benefit from ADM and only 30-40% achieve clinical remission after initial antidepressant treatment [4, 5]. Currently, in clinical practice, the selection of drugs for MDD is mainly relied on the measurements of symptoms and the experience of psychiatrists owing to lack of objective biomarkers for selecting antidepressants [4]. This “trial-and-error” approach takes approximately 4–8 weeks to find out whether antidepressant drugs are effective and then to develop the follow-up treatment program, which not only results in a lower response rate to subsequent medications and a waste of medical resources, but also prolongs rehabilitation time and increases suicide risk [4–7]. Therefore, it is of great significance for optimizing the way of drug selection if the response prediction of individualized treatment can be achieved prior to initial ADM [6, 7]. Previous studies suggested that early improvement in MDD signified clinical “turning point”, stable remission and good prognosis [8–11]. To clarify the earliest time point when improvement occurred, a meta-analysis and an observational study both stated that the response to ADM could occur as early as 2 weeks after initial ADM [10, 11]. With the help of early response prediction, psychiatrists can make decisions to continue or change treatment regimens earlier, especially to be able to cease ineffective treatment and reduce side effect and therapy-related risks. In the past few decades, numerous neuroimaging studies have focused on the exploration of the pathogenesis of MDD, while little is known about the pathophysiological substrate underlying the response of some patients to antidepressant medication while others with difficulty in rehabilitation [9–11].

At present, research with different neuroimaging modalities of magnetic resonance imaging (MRI) have identified several potential biomarkers associated with treatment response to specific therapies in MDD. For example, findings have shown that changes of cortex thickness and volume of gray matter (GM) in multiple brain regions including, but not limited to, the prefrontal cortex, hippocampus, anterior cingulate cortex at baseline can serve as imaging biomarkers to predict remission after medication and cognitive behavioral therapy (CBT) [12–14]. A recent study found that the anisotropy fraction (FA) of the white matter (WM), the most used metric for diffusion tensor imaging (DTI), could be utilized to predict antidepressant response. Increase of FA in the superior corona radiata and external capsule was correlating with drug response [15]. FA in the right amygdala, cingulate gyrus and terminal fasciculus could also predict antidepressant remission at 4–12 weeks [16–18]. One study identified the hippocampus and amygdala centered implicit emotion regulation circuitry as a sensitive biomarker in predicting early efficacy of SSRIs treatment [19]. FA tracts connecting hippocampus and amygdala have been reported to predict remission following SSRIs treatment [20]. The role of hippocampus in predicting 4-week SSRIs treatment response was also emphasized in functional research [21]. For SNRIs treatment, a neuroimaging study highlighted the importance of orbital superior frontal gyrus (ORBsup) and putamen centered neural circuitry as a biomarker [19]. Positron emission tomography / computed tomography (PET/CT) study also suggested that the activity of the anterior medial orbitofrontal cortex was associated with the remission of MDD after treatment with SNRIs [22, 23]. In addition, SSRI acts by blocking 5-hydroxytryptamine reuptake, whereas SNRI works by blocking the reuptake of 5-hydroxytryptamine, norepinephrine and dopamine at dendrites and axons, and the SSRI and SNRI neurofibrillary projection areas have been reported to be different [24]. All these evidence indicate that there may be some brain regions in a variety of spatially diverse GM and WM in associated with drug efficacy, so it is reasonable to explore imaging markers for predicting ADM response and assisting drug selection by combining multiscale structural imaging features of gray and white matter. Moreover, structural MRI (sMRI) is usually part of brain routine examination, and structural imaging data are easily available and relatively stable. However, most of the previous studies used only a single imaging modality and the results were often inconsistent and difficult to compare [12–17]. Also, these researches mainly focused on medium to long-term outcomes (4–12 weeks) [10, 11], with less involvement in predicting the earlier response to ADM in MDD patients.

Radiomics, a framework that combines machine learning with medical imaging to quantitatively reveal macroscopic heterogeneity that is unrecognizable to the human eyes, has greatly expanded the applications of conventional imaging in clinical practice [25, 26] and has been employed to investigate imaging biomarkers for neuropsychiatric disorders, such as Alzheimer’s disease [27], bipolar disorder [28] and attention deficit hyperactivity disorder [29]. By extracting and selecting high-weighted radiomics features, diagnostic models outperform those classifiers based on the routine imaging indicators, and the discriminant radiomics features can be used as potential markers. However, properties of MRI scanners, such as manufacturer, field strength, nonlinear gradient fields, and longitudinal drift, increase the bias and variability of brain sMRI [26, 30], thereby impacting the consistency and reproducibility of downstream analyses [31, 32], which will hinder the exploration of radiomics models and their transformation into diagnostic or predicting tools [30]. Like the “batch effect” in genomics, the term “scanner effect” is used in neuroimaging to refer to such abiotic variation [30]. Several methods have been proposed to harmonize CT and PET-CT conventional imaging indicators, such as hybrid white stripe and histogram matching, in previous studies on breast phantom [32], brain tumor [33], and prostate cancer [30]. Only ComBat technique has been validated in radiomics studies [31, 32], which could effectively address the issues of poor reproducibility and stability of the radiomics features.

To date, there has been few radiomics studies on the prediction of early improvement to ADM in adolescents with MDD based on harmonized multiscale sMRI [34]. As such, in present study, we constructed a prediction model for early improvement (2 weeks) to ADM in adolescent MDD patients with radiomics analysis after ComBat harmonization based on multiscale sMRI, including shape parameters of GM based on surface-based morphology (SBM) and voxel-based morphology (VBM) from the three-dimensional T1 weighted imaging (3D-T_1_WI), and diffusion properties of WM from the diffusion tensor imaging (DTI), and compared it with the model based on conventional imaging indicators. We also identified radiomics features that might be helpful for the objective first-line drugs selection of SSRIs and SNRIs in clinical practice.

## Methods

### Participants

138 patients with MDD, aged between 13 and 18 years, all right-handed according to their self-report, were prospectively and consecutively recruited from the Department of Psychiatry of the Second Affiliated Hospital of Kunming Medical University from December 2021 to June 2022. The study was approved by the Ethics Committee according to the principles of the Declaration of Helsinki with approval number KYCS202107. The written informed consent was obtained from participants and from their parents or legal guardians for subjects under 16 years old before enrollment. All participants were diagnosed by psychiatrists based on structured interviews and neuropsychological measurements and met the Diagnostic and Statistical Manual of Mental Disorders, Fifth Edition (DSM-V) criteria, including only unipolar and first-episode depression without any medication and physical therapy such as repetitive transcranial magnetic stimulation (rTMS) and electroconvulsive therapy (ECT) prior to hospitalization. 17 item Hamilton Depression Rating Scale (HAM-D_17_) was used to assess the severity of depressive symptoms at baseline and after 2-week ADM treatment. Exclusion criteria for MDD patients were: ①accompanied with any other mental disorders; ②any history of cranial injury; ③significant physical diseases undergoing treatment; ④substance/alcohol abuse or dependence; ⑤MRI scanning contraindicated.

All patients received SSRIs or SNRIs according to clinical guideline for the treatment of MDD patients (Chinese version) [5]. The doses were as follows: sertraline 100 ~ 200 mg/day, escitalopram 10 ~ 20 mg/day, fluvoxamine 100 ~ 300 mg/day, paroxetine 20 ~ 60 mg/day, fluoxetine 20 ~ 60 mg/day, venlafaxine 37.5 ~ 225 mg/day, and duloxetine 60 ~ 120 mg/day. HAM-D_17_ score was assessed again after 2 weeks of complete ADM. A case was defined as early improvement to ADM if the HAM-D_17_ score decreased ≥ 20% compared with the baseline [35]. 17 patients were excluded because they failed to complete 2-week ADM, including 13 cases of medication regimen change and 4 cases with additional rTMS. Finally, the remaining 121 MDD patients were divided into ADM improvers (SSRIs improvers and SNRIs improvers) and non-improvers according to reduction rate of HAM-D_17_ score.

### MRI protocol

**Fig. 1:**
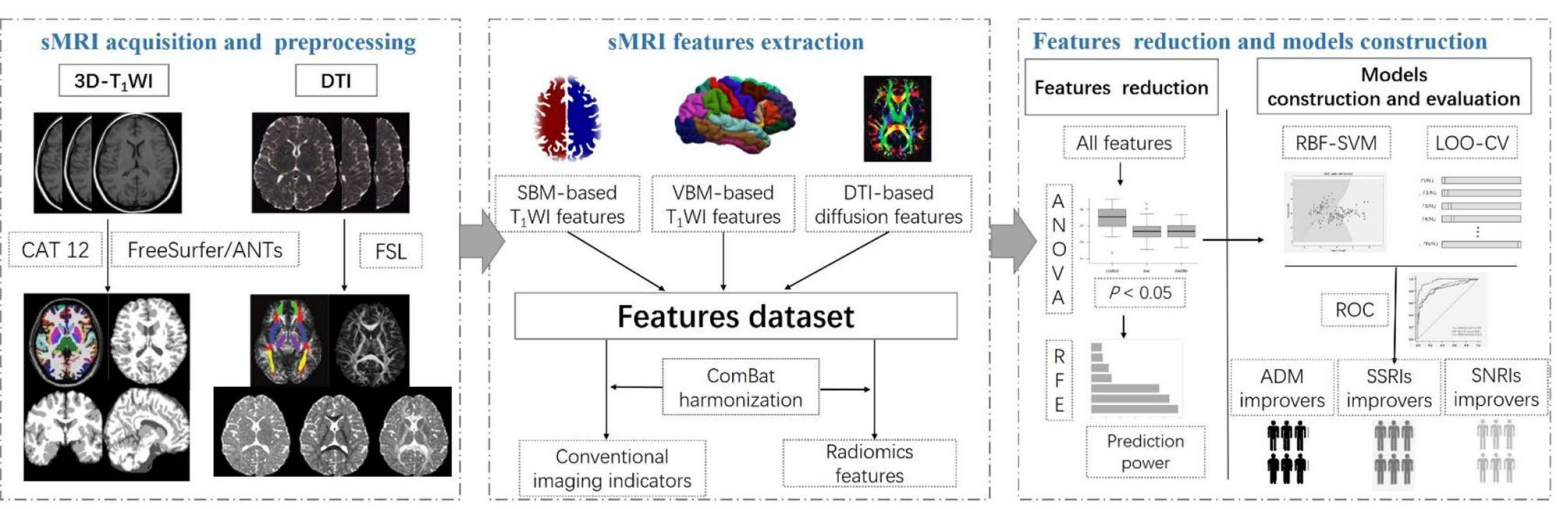
Flowchart of the study. Firstly, sMRI (3D-T_1_WI and DTI) was performed with two scanners in adolescent MDD subjects. FreeSurfer/ANTs hybrid segmentation toolkit of Mindboggle software, CAT 12 suite of SPM software and Diffusion Toolbox of FSL software were used for preprocessing. After that, conventional indicators and radiomics features of shape parameters of GM based on SBM and VBM analysis and diffusion properties of WM were extracted and then harmonized with ComBat technique. After receiving SSRIs or SNRIs for 2 weeks, the subjects were divided into ADM improvers (SSRIs improvers and SNRIs improvers) and non-improvers according to reduction rate of the HAM-D_17_ score. Finally, the features were decreased and filtered based on ANOVA and RFE successively and those with high prediction power were employed to construct models based on RBF-SVM. The performance was estimated using LOO-CV and ROC curve. sMRI, structural MRI. 3D-T_1_WI, three-dimensional T1 weighted imaging. DTI, diffusion tensor imaging. MDD, major depressive disorder. SPM, statistical parametric mapping. FSL, FMRIB’s software library. SBM, surface-based morphology. VBM, voxel-based morphology. GM, gray matter. WM, white matter. ANOVA, analysis of variance. RFE, recursive feature elimination. SVM, support vector mechanism. RBF, radial basis function kernel. LOO-CV, leave-one-out cross-validation. ROC, receiver operator characteristic. ADM, antidepressant medication. SSRIs, selective serotonin reuptake inhibitors. SNRIs, serotonin norepinephrine reuptake inhibitors. HAM-D_17_, Hamilton Depression Rating Scale, 17 item

The flowchart of this study is illustrated in Fig. 1. All participants underwent MRI scans within one week prior to ADM after structured interviews and neuropsychological measurements. 3D-T_1_WI, fluid attenuation inversion recovery (FLAIR), and DTI were performed sequentially using two scanners (Philip 3.0T, GE 3.0T), and the parameters of scanners and sequences are shown in Table S1 and Table S2. Examination of FLAIR was to rule out the presence of a substantial lesion, such as demyelination, brain tumor, vascular malformation, and development abnormalities. All images were reviewed immediately after each sequence completing, and those with motion artifacts needed to be rescanned and those with susceptibility artifacts were excluded. At last, one subject with excessive artifacts in sMRI was excluded. The final sMRI data of 63 subjects acquired at Philip 3.0T scanner and 58 subjects examined on GE 3.0T scanner were used for preprocessing, feature extraction and ComBat harmonization.

### SBM-based 3D-T_1_WI preprocessing and GM features extraction

3D-T_1_WI preprocessing based on SBM was accomplished using the FreeSurfer/ANTs hybrid segmentation pipeline [29] with the Desikan-Killiany-Tourville (DKT) atlas [36]. After performing the steps of motion correction, skull stripping, bias field correction, intensity normalization, expansion and smoothing, spherical mapping and alignment, and cortical surface reconstruction, the hybrid segmentation was achieved by combining the FreeSurfer recon-all toolkit (https://surfer.nmr.mgh.harvard.edu/) and the ANTsXNet CorticalThickness toolbox (http://stnava.github.io/ANTs/) introducing new gray-white matter boundaries to reduce segmentation errors, which has been proven to have superior performance over the FreeSurfer package [37]. Then, the following measures were automatically calculated using the feature extraction toolkit of Mindboggle (http://www.mindboggle.info/) [29] software: ①surface area of labeled brain; ②surface morphometric measures of cortical mesh vertex within labeled brain region and sulci; ③statistical metrics for each morphometric measure. In DKT atlas, there were thirty-eight cortical/subcortical regions and twenty-four sulci in each hemisphere. Therefore, based on the SBM method, 344 conventional indicators and 2,338 radiomics features representing GM surface morphometry were extracted from 3D-T_1_WI of each subject.

### VBM-based 3D-T_1_WI preprocessing and GM features extraction

3D-T_1_WI preprocessing based on VBM was performed using the CAT 12 toolkit [38] of the Statistical Parametric Mapping (SPM 12, https://www.fil.ion.ucl.ac.uk/spm/) software. The main steps were as follows: ①images were registered from the original brain space to a standard space coordinate system established by the Montreal Neurological Institute (MNI); ②bias field was estimated using the N4-Bias-Field-Correction algorithm [39] and then overlayed onto the original images to achieve bias field correction, thereby reducing the intensity difference of the same tissue and facilitating tissue segmentation; ③after skull stripping, the spatially normalized images were segmented into GM, WM, and cerebrospinal fluid (CSF). According to the Automated Anatomical Labeling Atlas (AAL) atlas, the preprocessed images of each subject were labeled into one hundred and sixteen brain regions, including ninety cerebral regions and twenty-six cerebellar regions. A neuroradiologist with 7-year experience of neuroimaging used ITK-SNAP 4.0 software (http://www.itksnap.org/pmwiki/) to examine and correct areas of brain tissue that were segmented incorrectly. Then, the segmented data were imported into LIFEx software (https://www.lifexsoft.org), and a total of sixty-three radiomics features of three categories, namely, five shape features, twelve first-order histogram features, and forty-six texture features were extracted from each brain label according to the image biomarkers standardization initiative (IBSI) guidelines [40] and radiomics quality scoring (RQS) tool [41]. Finally, based on the VBM analysis, 206 conventional indicators and 10,044 radiomics features representing GM density morphometry were extracted from 3D-T_1_WI images of each subject.

### DTI preprocessing and WM diffusion features extraction

DTI was preprocessed using Diffusion Toolbox v2.0 [42] of FMRIB’s Software Library (FSL, https://fsl.fmrib.ox.ac.uk/fsl/fslwiki/) for susceptibility-induced distortions, eddy-current and head motion correction, tensor fitting and registration to standard space. Diffusion tensors data from all subjects were used to build a template specific to this study by using an iterative tensor-based registration algorithm combined with local WM fiber bundle alignment. Each fraction map of the Johns Hopkins ICBM-DTI-81 white matter atlas was registered to the study-specific template through deformable alignment. Through nearest neighbor interpolation and alignment from each subject to the template, the label of the WM region in the map was aligned and warped into a single space, generating a partial anisotropic map, and labeling forty-eight WM regions. The following measures were computed, including the anisotropy fraction (FA), mean diffusivity (MD), axial diffusivity (AD), radial diffusivity (RD) and their corresponding statistical metrics for each WM label. Therefore, 192 conventional indicators and 768 radiomics features were extracted from each DTI data.

### ComBat harmonization

The extracted features were harmonized between the two scanners using the ComBat technique [31–33], which was designed to adjust any abiotic differences, i.e., scanner effects, that might be caused by the scanners, coils, and/or protocols parameters. The final ComBat-harmonized radiomics features were defined as:

$${Y}_{ijv}^{Combat}=\frac{{Y}_{ijv}-{\widehat{\alpha }}_{v}-{X}_{ij}{\widehat{\beta }}_{v}-{\gamma }_{iv}^{\text{*}}}{{\delta }_{iv}^{\text{*}}}+{\widehat{\alpha }}_{v}+{X}_{ij}{\widehat{\beta }}_{v}$$

In the above function, ${\widehat{\alpha }}_{v}$ is the overall value at voxel $v$, such as cortical thickness, volume, or FA value; ${X}_{ij}$ is the matrix of covariates (e.g., age, sex); ${\widehat{\beta }}_{v}$ represents the voxel-based regression coefficients corresponding to the $X$. ${\widehat{\alpha }}_{v}$ and ${\delta }_{iv}^{\text{*}}$ denote respectively the additivity and multiplication effects of site $i$ on voxel $v$. The above ComBat function assigned a specific transformation to each measure extracted from T_1_WI and DTI, respectively.

### Reduction of features

In order to eliminate extraneous features which affected the fitting efficiency and accuracy of models, a two-level selection strategy was used sequentially in this study. Firstly, analysis of variance (ANOVA) was applied to filter out features with *P* value less than 0.05. Then, recursive feature elimination (RFE) was applied to further reduce dimensionality of features by ranking them according to their relevance to each other and importance to the model in the reverse elimination process [43].

### Identification of radiomics features with high prediction power

In present study, because of small sample size, LOO-CV was used to validate the prediction model to avoid overfitting while being able to maximize the inclusion of samples during model training and testing. In each LOO-CV iteration of n samples, n-1 samples were used for training and one sample was left for testing. RFE process was conducted on the training dataset and selected the desired features by recursively and continuously reducing the size of the feature datasets. Each feature’s prediction power was estimated quantitatively by its weight vector, which was the distance from the feature to the separation hyperplane or decision boundary in the vector mapping space. Then, the prediction power in all LOO-CV iterations were averaged and ranked, and the top 30% were identified as the most powerful features.

### Prediction model construction and evaluation

When obtaining feature datasets with high prediction power, we used selected conventional indicators and harmonized radiomics features to construct support vector machine with radial basis function kernel (RBF-SVM) model on the LIBSVM toolbox (https://www.csie.ntu.edu.tw/~cjlin/libsvm) for prediction ADM non-improvers, SSRIs improvers and SNRIs improvers. All participants were labeled into three types (Label 1 = SSRIs-improver, Label 0 = ADM-non-improver, Label-1 = SNRIs-improver) to identify decision boundaries in the input space. To ensure the accuracy of parameter tuning, the grid search method was used to determine the optimal regularization parameter C and the kernel function parameter gamma for RBF-SVM model. After the tuning range and tuning step were given, the possible values of each parameter were calculated. Then all combination situations were traversed, and finally the best parameters were returned. Area under the curve (AUC), accuracy, sensitivity, and specificity based on LOO-CV results of the SVM were computed to evaluate the performance of the models. Permutation tests were used for assessing the generalization rate.

### Statistical analysis

Statistical analyses were conducted using R 3.5.1 software (Comprehensive R Archive Network). Quantitative data were summarized as the mean ± standard deviation or median with interquartile range, and categorical variables were described as numbers and percentages. ANOVA or Chi-square test was used for the univariate comparison as appropriate between the groups. AUC, sensitivity, and specificity were calculated by analysis of receiver operating characteristic (ROC) curve at cut-off score corresponding to the highest Youden index. Pairwise and multiple AUCs comparisons were performed using the DeLong tests. *P* value less than 0.05 was statistically significant.

## Results

### Demographic data and clinical information of participants

Results of demographic data and clinical information of participants are listed in Tables 1 and 2. There was no significant difference between ADM improvers and non-improvers, SSRIs improvers and SNRIs improvers in terms of age, gender, years of education, and baseline HAM-D_17_ score (*P* > 0.05).

**Table 1:** Demographic and clinical information of ADM improvers and non-improvers

Characteristics	ADM improvers (*n* = 67)	ADM non-improvers (*n* = 54)	*t*/*χ*2	*P*
Age (years)	15.0 ± 2.5	14.7 ± 2.0	-1.017	0.314
Gender (M/F)	31 / 36	24 / 30	0.128	0.722
Education (years)	9.5 ± 1.1	10.1 ± 0.5	0.637	0.530
Baseline HAM-D_17_ score	23.4 ± 4.5	22.7 ± 5.0	-0.672	0.511
2-week HAM-D_17_ score	14.9 ± 3.3	18.3 ± 4.7	4.330	0.000

*ADM* Antidepressant Medication, *MDD* Major Depressive Disorder, *HAM-D*_*17*_ 17-items of Hamilton Depression Rating Scale, *M* Male, *F* Female

**Table 2:** Demographic and clinical information of SSRIs improvers and SNRIs improvers

Characteristics	SSRIs improvers (*n* = 31)	SNRIs improvers (*n* = 36)	*t*/*χ*2	*P*
Age (years)	15.3 ± 2.3	15.2 ± 2.7	-0.440	0. 764
Gender (M/F)	15 / 16	16 / 20	1.322	0.082
Education (years)	9.9 ± 1.2	9.1 ± 1.7	-0.689	0.550
Baseline HAM-D_17_ score	25.4 ± 3.8	24.2 ± 3.5	-0.732	0.490
2-week HAM-D_17_ score	12.6 ± 2.1	13.0 ± 2.4	0.911	0.362

*SSRIs* Selective Serotonin Reuptake Inhibitors, *SNRIs* Serotonin Norepinephrine Reuptake Inhibitors, *HAM-D*_*17*_ 17-items of Hamilton Depression Rating Scale, *M* Male, *F* Female

### Features selection and identification of radiomics features with high prediction power

**Fig. 2:**
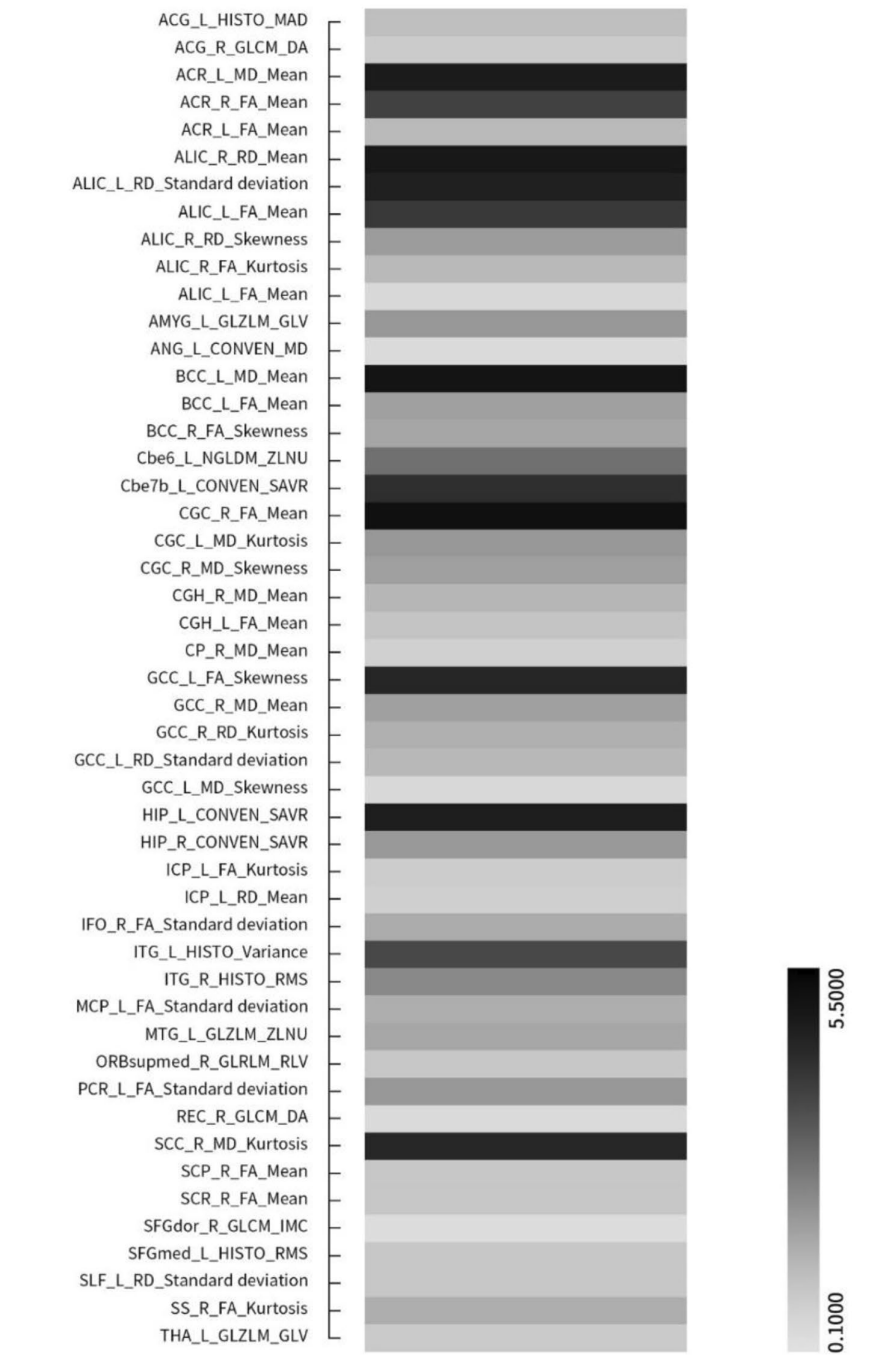
Prediction power of 49 radiomics features for predicting early improvement of MDD patients to ADM with SSRIs and SNRIs for 2 weeks. The grayscale bar represents the power, the darker the color, the greater the power; the lighter the color, the smaller the power. MDD, major depressive disorder. ADM, antidepressant medication. SSRIs, selective serotonin reuptake inhibitors. SNRIs, serotonin norepinephrine reuptake inhibitors

After preprocessing, a total of 742 conventional indicators (344 SBM-based features, 206 VBM-based features, and 192 DTI diffusion features) (Table S3) and 13,150 radiomics features (2,338 SBM-based features, 10,044 VBM-based features, and 768 DTI diffusion features) were extracted from 3D-T_1_WI and DTI (Table S4). These features were harmonized with ComBat technique. Then, the two-level dimensionality reduction strategy was performed and only 8 conventional indicators (2 VBM-based features, 6 diffusion features) and 49 radiomics features (16 VBM-based features, 33 diffusion features) were selected (Fig. 2).

**Fig. 3:**
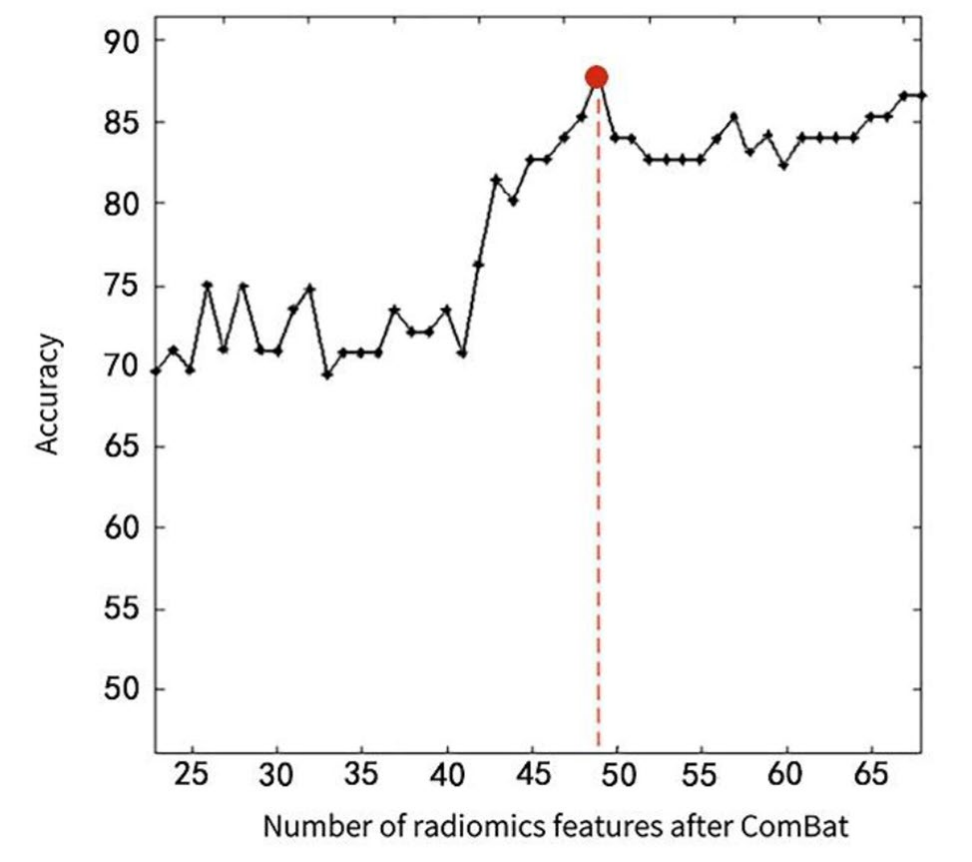
Learning curve for selection of ComBat-harmonized radiomics feature based on RFE. The X- / Y-axes represents the number of ComBat-harmonized radiomics features selected and the prediction accuracy, respectively. The highest overall accuracy (88.19%) of the radiomics prediction model was achieved when based on a minimum number (49) of radiomics features, and then the prediction performance did not improve as the number of features increased. RFE, recursive feature elimination

A 49-dimensional features dataset with different prediction power was obtained after each LOO-CV iteration and 121 49-dimensional feature datasets were achieved after 121 iterations, in which the best feature dataset was determined when the model reached its highest values after feature search completing (Fig. 3, Table S5). The prediction power in all LOO-CV iterations were averaged and ranked in descending order, and the top 30% were defined as the most powerful features for predicting ADM improvers, SSRIs improvers and SNRIs improvers. The radiomics features predicting ADM improver were mainly located in the hippocampus, medial orbitofrontal gyrus, anterior cingulate gyrus, amygdala, superior frontal gyrus, cerebellum (lobule vii-b), middle temporal gyrus, body of corpus callosum, anterior limb of internal capsule and anterior corona radiata. The radiomics features predicting SSRIs improver were primarily distributed in hippocampus, amygdala, inferior temporal gyrus, thalamus, cerebellum (lobule vi), fornix, and cerebellar peduncle. The radiomics features predicting SNRIs improver were primarily located in the medial orbitofrontal cortex, anterior cingulate gyrus, ventral striatum, accumbency area, knee of corpus callosum, internal capsule and anterior corona radiata.

### Prediction models performance

**Fig. 4:**
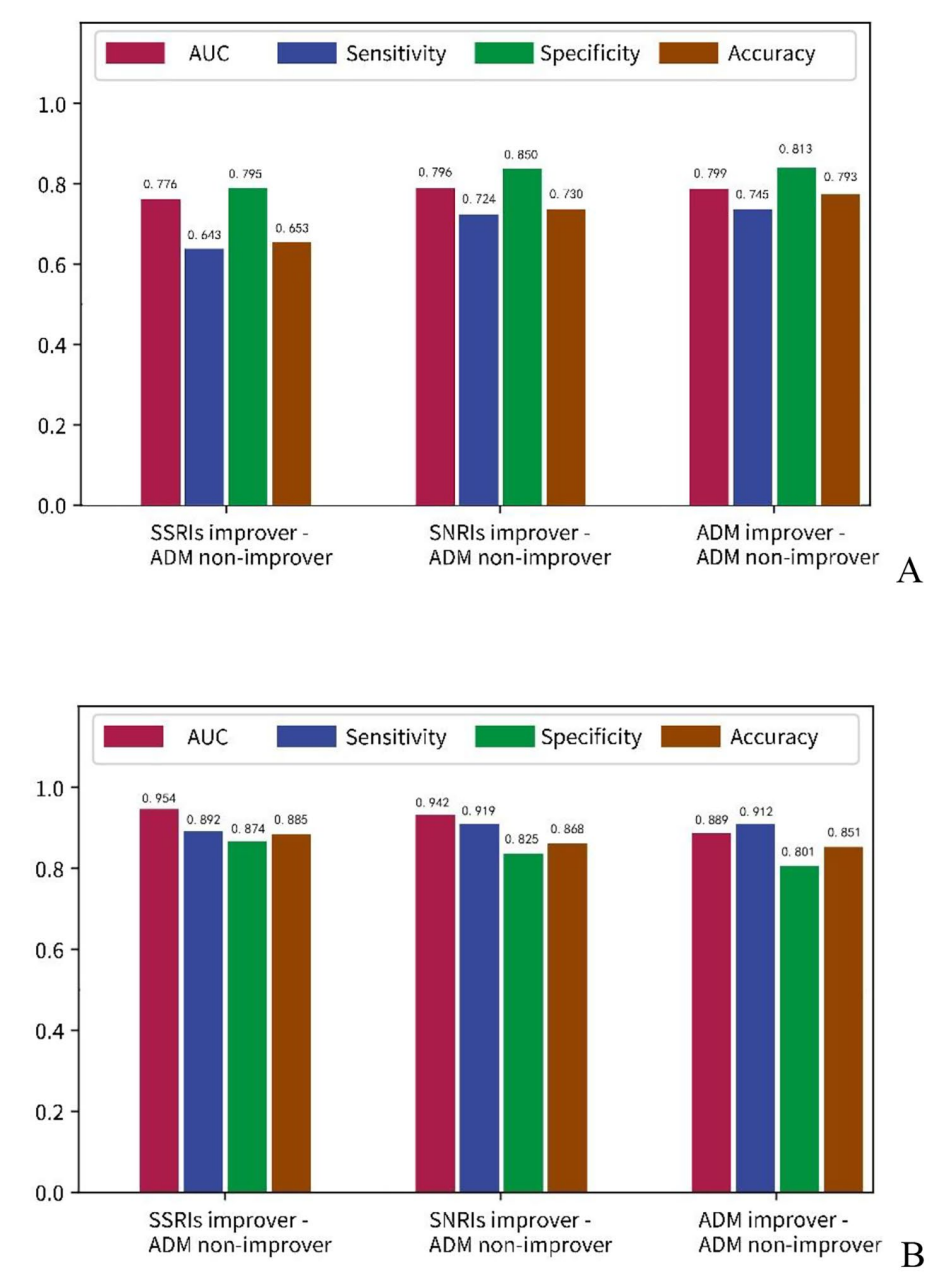
Performance of the SVM models predicting early improvement of MDD patients to ADM. (**A**) SVM model based on the conventional imaging indicators; (**B**) SVM model based on the ComBat-harmonized radiomics features

RBF-SVM models were constructed by using selected conventional imaging indicators and radiomics features. The optimal values of the regularization parameter C and the kernel function parameter gamma for RBF-SVM by the process of tuning and searching were 10^2^ and 10^− 1^, respectively. The detailed results of the SVM based on the two types of features are illustrated in Table 3. The overall accuracy of SVM based on conventional indicators and radiomics features after harmonization were 74.80% and 88.19%. The SVM model using ComBat-harmonized radiomics features based on the results of LOO-CV and ROC analysis had the better performance for predicting ADM improvers, SSRIs improvers and SNRIs improvers with the AUC, accuracy, sensitivity, and specificity of 0.889, 91.2%, 80.1% and 85.1%, 0.954, 89.2%, 87.4% and 88.5%, 0.942, 91.9%, 82.5% and 86.8%, respectively, which was superior to the model based on the conventional imaging indicators (Fig. 4). *P* value of permutation tests were less than 0.001.

**Table 3:** Performance of SVM prediction models

Models	AUC (95% CI)	Sensitivity (%)	Specificity (%)	Accuracy (%)
Model based on conventional indicators				
SSRIs improver - ADM non-improver	0.776(0.684, 0.853)	64.3(57.5, 71.9)	79.5(69.8, 82.0)	65.3(60.5, 76.8)
SNRIs improver - ADM non-improver	0.796(0.688, 0.882)	72.4(60.1, 79.9)	85.0(70.2, 89.7)	73.0(62.5, 86.1)
ADM improver -ADM non-improver	0.799(0.674, 0.872)	74.5(62.5, 83.2)	81.3(69.0, 88.4)	79.3(70.3, 89.4)
**Model based on radiomics features**				
SSRIs improver - ADM non-improver	0.954(0.912, 0.980)	89.2(78.8, 95.2)	87.4(79.4, 93.1)	88.5(82.4, 92.5)
SNRIs improver - ADM non-improver	0.942(0.897, 0.971)	91.9(83.2, 96.6)	82.5(73.8, 89.3)	86.8(80.5, 91.1)
ADM improver -ADM non-improver	0.889(0.798, 0.952)	91.2(76.8, 98.1)	80.1(64.4, 90.9)	85.1(75.0, 92.3)

*SVM* Support Vector Mechanism, *AUC* Area Under the Curve, *CI* Confidence Interval, *MDD* Major Depressive Disorder, *SSRIs* Selective Serotonin Reuptake Inhibitors, *SNRIs* Serotonin Norepinephrine Reuptake Inhibitors

## Discussion

Precision medicine is a long-term goal pursued in psychiatry clinics [44, 45]. Nevertheless, antidepressant drug selection is a complex and challenging issue in clinical practice [4, 5]. Gradually, neuroimaging studies based on radiomics and machine learning are demonstrating an important subsidiary role in the personalized diagnosis and treatment of psychiatric disorders [15–18, 27–29]. In this study, by integrating imaging features of brain multiscale sMRI after harmonization and selection, the radiomics model performed significantly better than the model based on the conventional imaging indicators, indicating that the integration and selection of high-dimensional features improved the predictive efficacy of the features, and the outstanding ability to select features was just the advantage of machine learning [25–27]. This radiomics framework confirmed that there were indeed differences in gray and white matter of important brain regions involved in early improvement to ADM of MDD patients by quantitatively estimating the power of each feature contributing to prediction model, which also made the results more interpretable.

The predictive ability of imaging markers represents the outcome of specific ADM agents to alleviate or eliminate structural or functional pathological changes in gray and white matter [25, 26]. The radiomics features identified in this study for the prediction of early ADM efficacy may help to choose SSRIs or SNRIs, thereby improving early response and long-term remission rates in patients with MDD. Features of the medial orbitofrontal gyrus, hippocampus, cerebellum lobule vii-b, corpus callosum, anterior limb of internal capsule and anterior corona radiata were extremely important in predictive models of 2-week ADM improvement. These brain regions are essential components of the brain’s emotion network and closely related to emotional processing, memory, and regulation [46, 47]. Previous studies have demonstrated that antidepressants could reverse impaired neuroplasticity and neurogenic changes in the hippocampus, such as loss of dendritic spines and synapses [48, 49]. According to the recent findings on hippocampal physiological function [50], the synergy between the hippocampus and other relevant brain regions at baseline could be integrated into a holistic feature to predict the beneficial effects of antidepressants on emotional memory and regulation. However, this needs to be further validated by neuropharmacological and psychological experiments after medication administration.

The present study showed that radiomics features of some brain regions for implicit emotion regulation such as hippocampus and amygdala play a prominent role in predicting 2-week improvement of SSRIs treatment. Several neurophysiological studies revealed that SSRIs had an acute neurological effect on emotion regulation by affecting 5-hydroxytryptamine reuptake and thus increasing 5-hydroxytryptamine concentrations in the synaptic space to regulate activity in relevant brain regions, especially in the hippocampus and amygdala [50–52]. There were also imaging studies showing that FA values of the WM fiber tract conjoining hippocampus and amygdala could predict long-term remission after receiving SSRIs [53], and the fornix connecting the hippocampus was an important predictor of early response to SSRIs [16, 17]. SSRIs are known to mediate hippocampal responsiveness to treatment by increasing neural progenitor cells, and a functional MRI study predicting 4-week efficacy also highlighted the hippocampal response to SSRIs therapy [54]. As for the amygdala, one study showed higher FA values in the amygdala of remitters compared to non-remitters after treatment with SSRIs [15, 16], which might be related to the 5-hydroxytryptamine-mediated amygdala response pattern as a key pathway for the antidepressant effects of SSRIs. MDD is a kind of psychiatric disorder with impaired emotion regulation, and habitual use of emotion regulation strategies plays an important role in MDD episodes [46, 47]. Therefore, based on our results and the known mechanisms of action of SSRIs, it is reasonable to use imaging features of emotional regulation-localized brain regions as markers to predict the early efficacy of SSRIs in MDD patients.

The present study also identified the radiomics features of some brain regions such as the medial orbitofrontal cortex, anterior cingulate gyrus, ventral striatum, accumbency area, knee of corpus callosum, internal capsule and anterior corona radiata as imaging markers for 2-week improvement of SNRIs. This was consistent with the results of previous neuropharmacological studies showing that the brain regions associated with SNRIs response were mainly located in the emotional regulation and reward circuits [50, 52]. The medial orbitofrontal cortex and anterior cingulate gyrus are involved in learning, motivation, and reward behavior in MDD patients [55, 56], while the ventral striatum is engaged in reward signaling in the neural system, and abnormalities in these regions can lead to anhedonia [46, 55–57]. SNRIs increase activity in the medial orbitofrontal cortex and ventral striatum by affecting norepinephrine reuptake and dopamine release to alleviate the pleasure deficit in MDD patients [52]. Positron emission tomography (PET) study also suggested that the activity of the anterior medial orbitofrontal cortex was associated with the remission of MDD after treatment with SNRIs [22, 23]. Meanwhile, our study indicated that DTI-related diffusion features of white matter in brain regions associated with emotional regulation and reward circuits were important biomarkers for predicting early improvement to SNRIs. Therefore, the results of this study were consistent with the above theoretical perspective.

Previous neuroimaging studies revealed that morphological features of the brain surface based on SBM analysis had a high weight in the diagnostic model of MDD [34] and could be used as predictive markers of response to antidepressant treatment [12–14]. For example, structural abnormalities accompanied by decreased functional connectivity at baseline in the hippocampus, superior frontal gyrus, middle temporal gyrus, cingulate gyrus, and amygdala associated with processing emotional processing were better predictors of antidepressant response [12–14, 16, 17]. However, the results of the present study showed that none of SBM-based features were selected in the radiomics prediction model, which might be related to the selecting strategy of imaging features by ranking them according to their relevance to each other and importance to the model in the reverse elimination process during machine learning, so the relationship between SBM-based imaging indicators and MDD drug efficacy needs to be further investigated.

Several limitations are certainly needed to be considered in our study. First, due to small sample size, we used the leave-one-out cross-validation and failed to use external samples for validation, which might affect the reliability of the prediction model. Second, the brain multiscale sMRI were obtained using different scanners, and although the radiomics features were harmonized by using the ComBat technique, it might also affect the model prediction performance. Third, the MDD patients were simply grouped using only scale scores and medication types, without considering the heterogeneity associated with different subtypes of depression and various biological entities, which may affect the effectiveness of the model.

## Conclusions

In summary, our findings suggest that the radiomics model based on brain multiscale sMRI after ComBat harmonization could effectively predict the early improvement of ADM in adolescent MDD patients with a high accuracy, which was superior to the model based on the conventional imaging indicators. The radiomics features with high prediction power may help for the individual SSRIs and SNRIs selection.

## Electronic supplementary material

Below is the link to the electronic supplementary material.

**Supplementary Material 1:** Radiomics features and parameters of MR scanners.

## Data Availability

The data used and analyzed in this study are available from the corresponding author on reasonable request.

## List of abbreviations

MDD: Major depressive disorder
ADM: Antidepressant medication
sMRI: Structural magnetic resonance imaging
SSRIs: Selective serotonin reuptake inhibitors
SNRIs: Serotonin norepinephrine reuptake inhibitors
3D-T_1_WI: Three-dimensional T1 weighted imaging
DTI: Diffusion tensor imaging
GM: Gray matter
WM: White matter
CSF: Cerebrospinal fluid
SBM: Surface-based morphology
VBM: Voxel-based morphology
ANOVA: Analysis of variance
RFE: Recursive feature elimination
SVM: Support vector machine
RBF: Radial basis function kernel
ROC: Receiver operating characteristic
AUC: Area under the curve
LOO-CV: Leave-one-out cross-validation
HAMD: Hamilton depression rating scale
DSM-V: Diagnostic and statistical manual of mental disorders, fifth edition
rTMS: Repetitive transcranial magnetic stimulation
ECT: Electroconvulsive therapy
FLAIR: Fluid attenuation inversion recovery
DKT: Desikan-Killiany-Tourville
SPM: Statistical parametric mapping
AAL: Automated anatomical labeling atlas
IBSI: Image biomarkers standardization initiative
RQS: Radiomics quality scoring
FSL: FMRIB’s software library
FA: Anisotropy fraction
