# Supplementary material for: Prediction of early improvement of major depressive disorder to antidepressant medication in adolescents with radiomics analysis after ComBat harmonization based on multiscale structural MRI
Source: BMC Psychiatry. 2023 Jun 26;23:466. doi: 10.1186/s12888-023-04966-8 (PMC10294484; doi:10.1186/s12888-023-04966-8)
Supplement: Supplementary file 1 — Supplementary Material 1: Radiomics features and parameters of MR scanners. [file 12888_2023_4966_MOESM1_ESM.docx]

**Table S1** Equipment parameters of MR scanners

| Parameters | MRI1 | MRI2 |
| --- | --- | --- |
| Manufacturer | Philips Medical Systems Koninklijke Philips N.V. | GE Healthcare，Waukesha，WI，USA |
| Model | Elition | Pioneer |
| Magnetic field strength (T) | 3.0 | 3.0 |
| Coil | 32-channel phased-array head coil | 21-channel phased-array head coil |

**Table S2** Acquisition parameters of 3D-T_1_WI and DTI

| Parameters | 3D-T_1_WI | | DTI | |
| --- | --- | --- | --- | --- |
|  | MRI1 | MRI2 | MRI1 | MRI2 |
| Sequence name | TFE | BRAVO | SE-EPI | SE-EPI |
| TR (ms) | 6.54 | 8.62 | 7200~7400 | 7200~7300 |
| TE (ms) | 2.94 | 3.34 | 95~105 | 98~105 |
| FOV (mm×mm) | 240×240 | 256×256 | 240×240 | 240×240 |
| Slice thickness (mm) | 1 | 1 | 3 | 3 |
| Spacing (mm) | 0 | 0 | 0 | 0 |
| Number of slices | 170 | 320-340 | 128 | 128 |
| Matrix | 256×256 | 256×256 | 130×128 | 130×128 |
| Voxel size (mm×mm×mm) | 0.9×0.9×1 | 0.5×0.5×0.5 | 1.6×1.8×3.0 | 1.6×1.8×3.0 |
| Directions | NA | NA | 64 | 64 |
| *b*-Values（sec/mm^2^） | NA | NA | 0/1000 | 0/1000 |

MRI1 - Philips 3.0T. MRI2 - GE 3.0T. 3D-T_1_WI - three-dimensional T1 weighted image. DTI - diffusion tensor imaging. TR - repetition time. TE - echo time. FOV - field of view. TFE - turbo field echo. BRAVO - brain volume imaging. SE-EPI - spin-echo echo-planar imaging sequence. NA - not applicable.

**Table S3** Types and numbers of conventional imaging indicators of sMRI

| Features source | Brain regions | Type of features | Number of features |
| --- | --- | --- | --- |
| 3D-T_1_WI based on SBM analysis of GM (DKT atlas) | Cortical regions  (31×2) | Surface area  Mean curvature  Convexity | 186（31×2×3） |
|  | Subcortical regions (7×2) | Volume | 14（7×2×1） |
|  | Sulci  (24×2) | Local cortical thickness  Mean curvature  Convexity | 144（24×2×3） |
|  | Subtotal | | 344 |
|  |  |  |  |
| 3D-T_1_WI based on VBM analysis of GM (AAL atlas) | Cerebral regions  (90) | Volume  Cortical thickness | 180（90×2） |
|  | Cerebellar regions  (26) | Volume | 26（26×1） |
|  | Subtotal | | 206 |
|  |  |  |  |
| DTI-based diffusion properties of WM | White matter regions（48） | FA  MD  AD  RD | 192（48×4） |
|  | Subtotal | | 192 |
|  | | |  |
| Total | | | 742 |

3D-T_1_WI - three-dimensional T1 weighted image. DTI - diffusion tensor imaging. DKT - Desikan-Killiany-Tourville. AAL - automated anatomical labeling atlas. SBM - surface-based morphology. VBM - voxel-based morphology. GM - gray matter. WM - white matter. FA - fraction anisotropy. MD - mean diffusivity. AD - axial diffusivity. RD - radial diffusivity.

**Table S4** Types and numbers of radiomics features of sMRI

| Features source | Brain regions | Type of features | | Number of features |
| --- | --- | --- | --- | --- |
| 3D-T_1_WI based on SBM analysis of GM (DKT atlas) | Cortical regions  (31×2) | Volume  Surface area  Local cortical thickness  Mean curvature  Convexity  Geodesic depth  Travel depth | | 2,338 |
|  | Subcortical regions  (7×2) |  |  |  |
|  | Sulci  (24×2) |  |  |  |
|  |  |  | |  |
| 3D-T_1_WI based on VBM analysis of GM (AAL atlas) | Cerebral regions  (90) | CONVEN-  HISTO-  GLCM-  GLRLM-  GLZLM-  NGLDM- | | 10,044 |
|  | Cerebellar regions  (26) |  |  |  |
|  |  |  | |  |
| DTI-based diffusion properties of WM | White matter regions  (48) | FA  MD  AD  RD | Mean  Standard deviation  Skewness  Kurtosis | 768 |
|  |  |  |  |  |
| Total | | | | 13,150 |

3D-T_1_WI - three-dimensional T1 weighted image. DTI - diffusion tensor imaging. DKT - Desikan-Killiany-Tourville. AAL - automated anatomical labeling atlas. SBM - surface-based morphology. VBM - voxel-based morphology. GM - gray matter. WM - white matter. FA - fraction anisotropy. MD - mean diffusivity. AD - axial diffusivity. RD - radial diffusivity.

**Table S5** Radiomics features for predicting early improvement to ADM of MDD patients

| Hemisphere | Brain region | Type of feature | Detailed feature | Power |
| --- | --- | --- | --- | --- |
| **DTI-based diffusion features (33)** | | | | |
| R | CGC | FA | Mean | 5.2417 |
| L | BCC | MD | Mean | 5.0003 |
| R | ALIC | RD | Mean | 4.9361 |
| L | ACR | MD | Mean | 4.8635 |
| L | ALIC | RD | Standard deviation | 4.7330 |
| L | GCC | FA | Skewness | 4.4464 |
| R | SCC | MD | Kurtosis | 4.3781 |
| L | ALIC | FA | Mean | 3.9154 |
| R | ACR | FA | Mean | 3.6262 |
| L | PCR | FA | Standard deviation | 1.9189 |
| R | ALIC | RD | Skewness | 1.9022 |
| L | CGC | MD | Kurtosis | 1.9233 |
| R | CGC | MD | Skewness | 1.8120 |
| L | BCC | FA | Mean | 1.8000 |
| R | GCC | MD | Mean | 1.7240 |
| R | BCC | FA | Skewness | 1.6533 |
| R | IFO | FA | Standard deviation | 1.4880 |
| R | SS | FA | Kurtosis | 1.3964 |
| R | CGH | MD | Mean | 1.3785 |
| L | MCP | FA | Standard deviation | 1.3177 |
| R | GCC | RD | Kurtosis | 1.3130 |
| R | ALIC | FA | Kurtosis | 1.1856 |
| L | ACR | FA | Mean | 1.1522 |
| L | GCC | RD | Standard deviation | 1.1034 |
| R | SCP | FA | Mean | 0.9642 |
| R | SCR | FA | Mean | 0.9327 |
| L | CGH | FA | Mean | 0.9289 |
| L | SLF | RD | Standard deviation | 0.8655 |
| L | ICP | FA | Kurtosis | 0.7440 |
| L | ICP | RD | Mean | 0.6514 |
| R | CP | MD | Mean | 0.5378 |
| L | ALIC | FA | Mean | 0.3770 |
| L | GCC | MD | Skewness | 0.3321 |
| **VBM-based features of 3D-T_1_WI (16)** | | | | |
| L | HIP | CONVEN_ | SAVR | 4.5803 |
| L | Cbe7b | CONVEN_ | SAVR | 4.0240 |
| L | ITG | HISTO_ | Variance | 3.3416 |
| L | Cbe6 | NGLDM_ | ZLNU | 2.7410 |
| R | ITG | HISTO_ | RMS | 2.1107 |
| L | AMYG | GLZLM_ | GLV | 1.9588 |
| R | HIP | CONVEN_ | SAVR | 1.9415 |
| L | THA | GLZLM_ | GLV | 1.6530 |
| L | MTG | GLZLM_ | ZLNU | 1.5309 |
| L | ACG | HISTO_ | MAD | 1.0004 |
| R | ORBsupmed | GLRLM_ | RLV | 0.9916 |
| L | SFGmed | HISTO_ | RMS | 0.8518 |
| R | ACG | GLCM_ | DA | 0.7506 |
| R | SFGdor | GLCM_ | IMC | 0.2390 |
| L | ANG | CONVEN_ | MD | 0.2042 |
| R | REC | GLCM_ | DA | 0.2018 |

*ADM* Antidepressant Medication, *MDD* Major Depressive Disorder, *DTI* Diffusion Tensor Imaging, *R* Right, *L* Left
